# Adaptation of the Recent Life Changes Questionnaire (RLCQ) to measure stressful life events in adults residing in an urban megapolis in Pakistan

**DOI:** 10.1186/s12888-017-1315-1

**Published:** 2017-05-05

**Authors:** Azmina Artani, Shireen Shehzad Bhamani, Iqbal Azam, Moiz AbdulSultan, Adeel Khoja, Ayeesha K. Kamal

**Affiliations:** 1 Stroke Service, The International Cerebrovascular Translational Clinical Research Training Program (Fogarty International Center, National Institutes of Health) and Aga Khan University, Karachi, Pakistan; 2 Aga Khan University School of Nursing & Midwifery, Karachi, Pakistan; 3 Biostatistics and Epidemiology, Department of Community Health Sciences, Aga Khan University, Karachi, Pakistan; 4 MBBS Program, Jinnah Medical and Dental College, Karachi, Pakistan; 5 Stroke Service, Department of Medicine, Aga Khan University, Karachi, Pakistan; 6 Section of Neurology, Department of Medicine, Stroke Fellowship Program, International Cerebrovascular Translational Clinical Research Training Program, Fogarty International Center, National Institute of Neurologic Disorders and Stroke (USA), Aga Khan University, Stadium Road, Karachi, 74800 Pakistan

**Keywords:** Recent life changes questionnaire, Adaptation, Pakistan

## Abstract

**Background:**

Contextually relevant stressful life events are integral to the quantification of stress. None such measures have been adapted for the Pakistani population.

**Methods:**

The RLCQ developed by Richard Rahe measures stress of an individual through recording the experience of life changing events. We used qualitative methodology in order to identify contextually relevant stressors in an open ended format, using serial in-depth interviews until thematic saturation of reported stressful life events was achieved. In our next phase of adaptation, our objective was to scale each item on the questionnaire, so as to weigh each of these identified events, in terms of severity of stress. This scaling exercise was performed on 200 random participants residing in the four communities of Karachi namely Kharadar, Dhorajee, Gulshan and Garden. For analysis of the scaled tool, exploratory factor analysis was used to inform structuring.

Finally, to complete the process of adaption, content and face validity exercises were performed. Content validity by subject expert review and face validity was performed by translation and back translation of the adapted RLCQ. This yielded our final adapted tool.

**Results:**

Stressful life events emerging from the qualitative phase of the study reflect daily life stressors arising from the unstable socio-political environment. Some such events were public harassment, robbery/theft, missed life opportunities due to nepotism, extortion and threats, being a victim of state sponsored brutality, lack of electricity, water, sanitation, fuel, destruction due to natural disasters and direct or media based exposure to suicide bombing in the city. Personal or societal based relevant stressors included male child preference, having an unmarried middle aged daughter, lack of empowerment and respect reported by females. The finally adapted RLCQ incorporated “Environmental Stress” as a new category.

**Conclusion:**

The processes of qualitative methodology, in depth interview, community based scaling and face and content validity yielded an adapted RLCQ that represents contextually relevant life stress for adults residing in urban Pakistan.

**Trial registration:**

Clinicaltrials.gov NCT02356263. Registered January 28, 2015. (Observational Study Only).

**Electronic supplementary material:**

The online version of this article (doi:10.1186/s12888-017-1315-1) contains supplementary material, which is available to authorized users.

## Background

Stress is defined as the loss of equipoise between evolving changes in an individual’s life and their capacity to adapt [1]. Although stress is a common phenomenon, its extent varies among people based on their perceptions and availability of appropriate coping strategies [2].

Assessing the burden of stress is not an easy task due to its subjectivity. As stress translates into many common mental disorders, the prevalence of mental disorders can serve as a relatively accurate measure for evaluating the effects of stress. Chronic stress may ultimately become the tipping point of common mental disorders such as depression, anxiety, mood disorders and suicide [3].

The Global Burden of Disease Survey conducted by World Health Organization (WHO) reports psychiatric illnesses along with stress-related disorders to be the second leading cause of disabilities by the year 2020 [4]. In 2010, depressive disorders had affected 40% of world’s population [5]. In Pakistan, studies from both rural and urban areas report prevalence of depression from 3.4% to 40% [6, 7].

Studies from Pakistan have reported tools that have been developed or adapted, translated and validated to measure stress [8]. These tools have been constructed for a specific condition e.g. anxiety or depression or for specific population for e.g. antenatal clinics, in-patient and out-patient psychiatric settings, adolescents and teachers (Table 1) [8–16]. There is a need for a generalizable tool to be used in community settings that can measure stress in the general population.

**Table 1:** Validated Stress Measurement Scales in Pakistan

Scale	Purpose	Validated against	Year (sample size) site	Strength	Population
General Health Questionnaire [GHQ-28] [1]	Translation and Validation	Hospital Anxiety and Depression Scale (HADS) by Zigmond (1983)	1998 (162) Karachi	Correlation coefficient *r* = 0.62 Cronbach’s α = 0.90	Medical and nursing students
Teacher’s stress inventory [TSI] [10]	Translation and adaptation	Original TSI by Fimian (1984)	2003 (120) Rawalpindi, Islamabad and Chakwal.	----	Teachers only
Aga Khan University Anxiety and Depression Scale [AKUADS] [3]	Development and validation	Psychiatric evaluation based on DSM-IV criteria	2005 (200) Hyderabad	ROC area under the curve = 0.73 Cronbach’s α = 0.87	Pregnant women only
How do I feel questionnaire [11]	Translation and validation	Psychiatric evaluation based on DSM-IV criteria	2005 (200) Hyderabad	ROC area under the curve = 0.74 Cronbach’s α = 0.92	Pregnant women only
The Pakistan Anxiety and Depression Scale [PADQ] [12]	Development and validation	Psychiatric evaluation based on DSM-IV	2005 (330) Lahore and Peshawar	Sensitivity = 91% Specificity = 94% Cronbach’s α = 0.92	In-patient and outpatient psychiatry patients.
Personal Health Questionnaire [PHQ] [13]	Translation and validation in community setting	Computer mediated interviews based on Psychiatric Assessment Schedule	2006 (267) Islamabad	Sensitivity = 78.9% Specificity = 85.2%	Validated for the construct of depression only
Self-Reporting Questionnaire [SRQ] [13]	Translation and validation in community setting	Psychiatric Assessment Schedule (PAS)	2006 (267) Islamabad	Sensitivity = 93.1% Specificity = 80.8%	Validated for the construct of depression only
A-Z Stress Scale [14]	Development and validation	Centre for Epidemiological Studies- Depression Scale (CES-D)	2009 (421) Karachi	Correlation coefficient *r* = 0.56 Cronbach’s α = 0.82 (one week)	Pregnant women only
Depression Scale for Adolescent Schoolgirls [DSAG] [15]	Development and validation	Children Depression Inventory (CDI) by Kovacs (2007)	2010 (587) Lahore	Correlation coefficient *r* = 0.68 Cronbach’s α = 0.88	Adolescent schoolgirls
School Children Problem Scale [SCPS] [16]	Development and validation	Youth Self Report (YSR)	2011 (853) Lahore	Correlation coefficient *r* = 0.7 Cronbach’s α = 0.79 (one week)	School children only (8th – 10th graders)
Daily Stressor Scale [DSS] [25]	Development and validation	Perceived Stress Scale (PSS) by Cohen (1983)	2012 (264) Pakistan	Correlation coefficient *r* = 0.46	University teachers

An important mediator of stress is the occurrence of repeated Stressful Life Events in the domains of home, health, work, environment and personal and social life. Rahe and Holmes developed a tool using these events so as to measure stress which is known as “*Social Readjustment and Rating Scale (SRRS)*”. However, SRRS had a limited item range which was reviewed periodically by these researchers and led to the development of another tool namely “Recent Life Changes Questionnaire (RLCQ)” in 1997 [17, 18]. RLCQ measures stress through life changing events occurring during a specified time frame (6 months or a year) and measures its consequences on health of an individual.

Pakistan is a developing country where daily life stressors have a major influence on mental health of people and no tools are available that measure stress in the general population in a relevant and contextually appropriate fashion. Hence, the aim of this study is to report the process of adaptation of the RLCQ with respect to the general population of urban Pakistan where the adapted tool becomes accurate and relevant.

## Methods

### Study design and setting

**Fig. 1:**
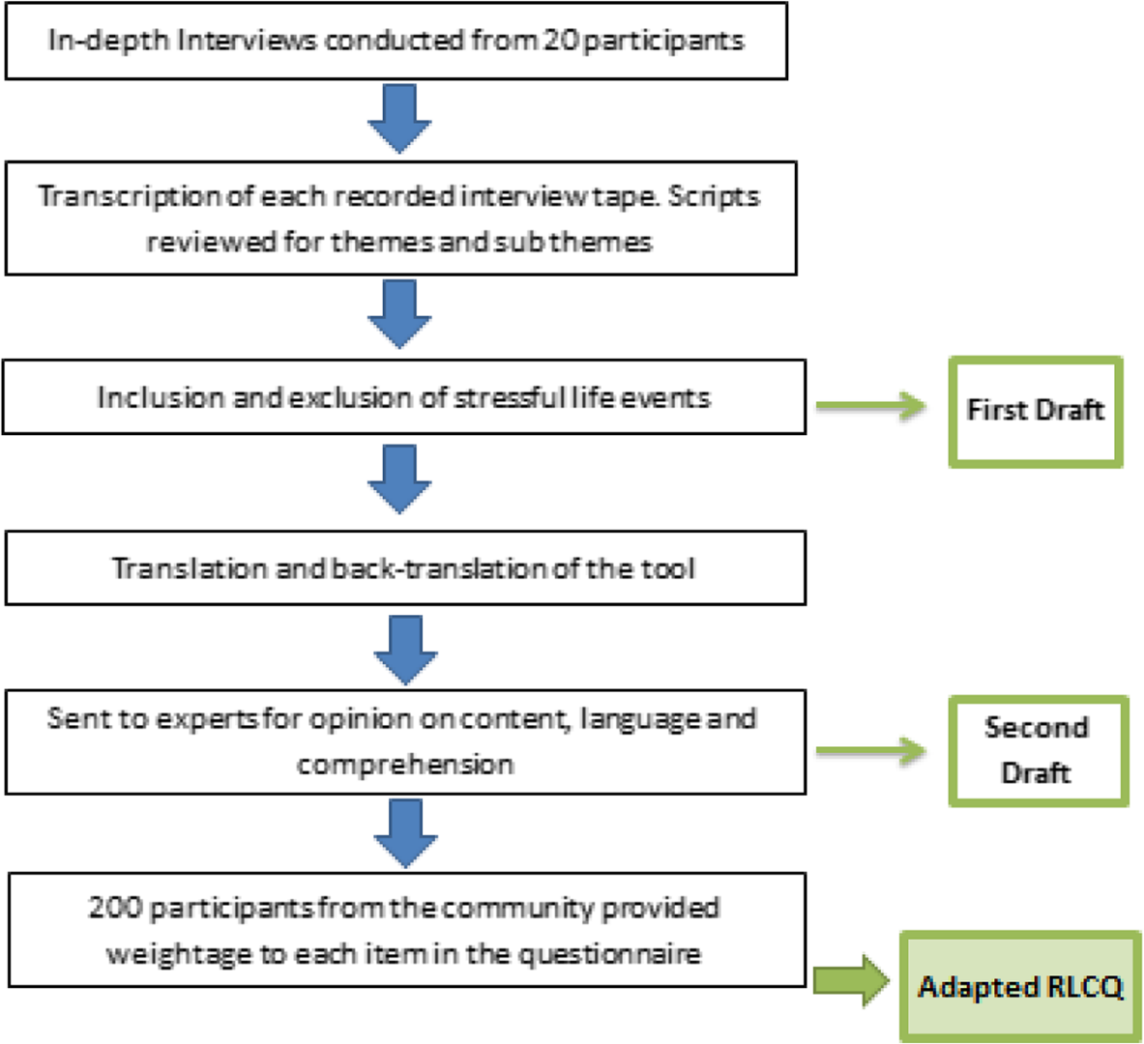
Study Flow Diagram

The study incorporated qualitative methodology following COREQ guidelines [19]. First, it followed a phenomenology study design as we aimed to explore stressors relevant to Pakistani context. We conducted serial in-depth interviews along with translating the tool and seeking expert’s opinion [19]. This was followed by community based scoring of each stressful item on the adapted tool (Fig. 1).

Karachi best represents the multi-ethnicity of Pakistan due to its metropolitan nature and extreme diversity and thus was the ideal site for this study for reasons of external validity.

### Populations for in-depth interviews and rating

Participants were recruited from the outpatient departments of Aga Khan University, Hospital (AKUH) which serves people from diverse socio-cultural backgrounds who visit for regular health checkups e.g. vaccination, eye checkups, employee assessment. They were invited and consented for in-depth interviews for theme identification of stressors that may not have been listed in the original RLCQ due to cultural differences.

**Fig. 2:**
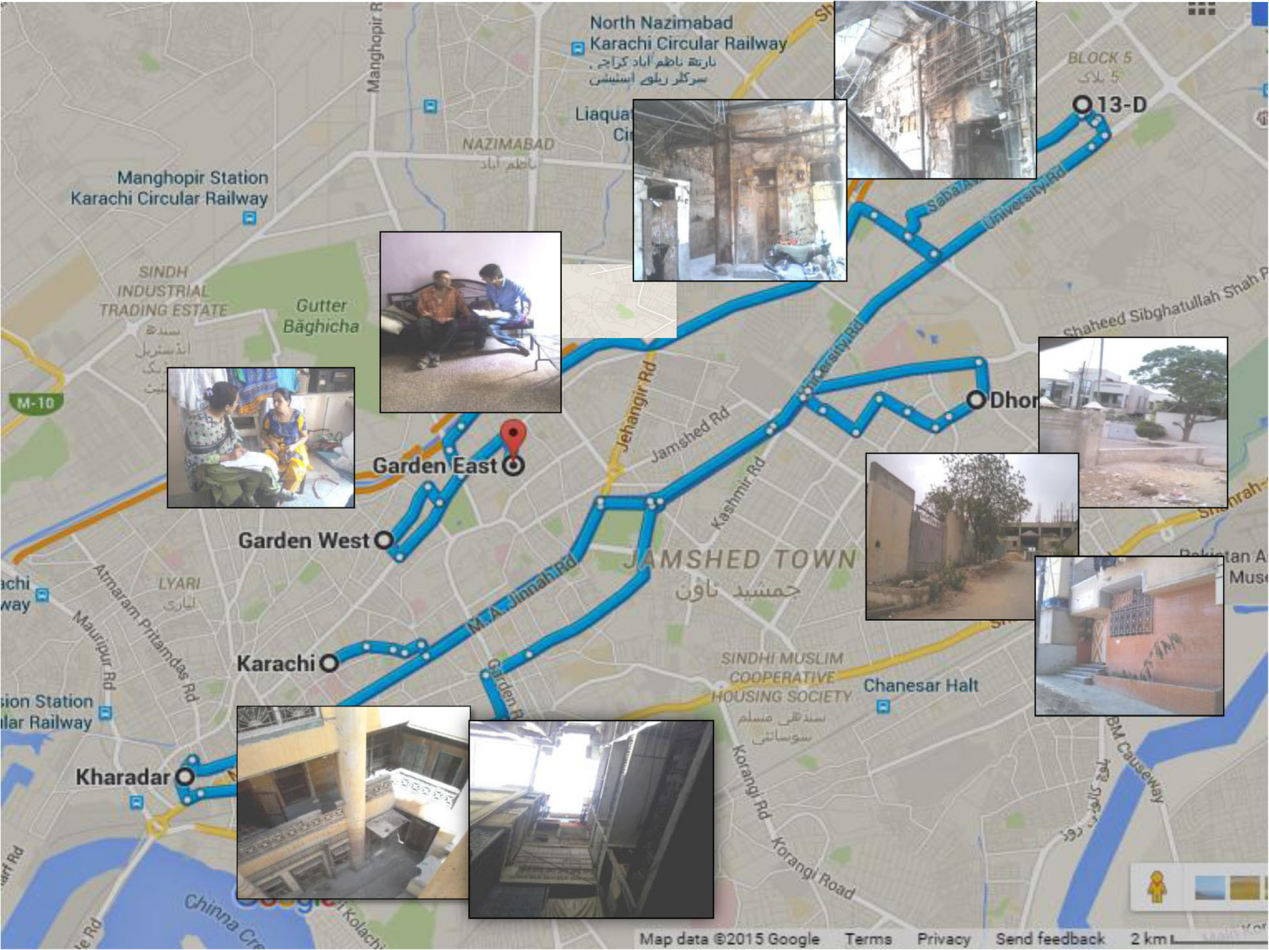
Community sites - Garden, Kharadar, Gulshan and Dhorajee

Following these open ended in-depth interviews and theme identification, we proceeded to community scaling. From February to March 2015, we recruited adult participants from four communities in Karachi namely Kharadar, Dhorajee, Gulshan and Garden. These communities represent the various ethnic groups within Pakistan and ensure a better generalization (Fig. 2). Those who could understand Urdu and were willing to participate were invited to be part of the study however; individuals who had been taking medications prescribed for existing mental conditions were excluded because the use of medication may have altered their perception of severity of daily life stressors. These communities helped rate the scale of stressful life events that were identified in the open ended qualitative part of the study.

### Study procedures

#### Step I: In-depth interviews and adaptation steps

Qualitative interviews began in December 2014 from men and women aged ≥18 years who were able to understand Urdu and provided written informed consent. We excluded those participants who had cognitive, hearing or speech difficulties due to strokes or other organic impairments. There was no direct personal relationship with the participants prior to study commencement. However, the community trusts and has a good relationship with the institution. The purpose of these interviews was to explore the understanding of stress and to gain insight into stressful life events at the community level. Respondents were purposefully identified based on their willingness to give us an extended length of time and to ensure participation of a diverse population such as representativeness of both males and females and of different sociocultural backgrounds. Participants were approached face to face and were informed about the researcher’s credentials, personal goals and the purpose of the study while obtaining informed consent.

AA conducted all the interviews; she is a qualified nurse and has training experience in qualitative research methods courses as an academic program. In addition she is a Clinical Research Fellow and Masters in Epidemiology and Biostatistics.

From initial recruitment, we continued to interview participants until same stressful events were being reported and no new stressful event was discussed with the interviewer. Sample size was based on the data saturation. We continued to interview participants until 20 participants were interviewed and theme saturation was achieved. The interviews were conducted in separate rooms that were identified at each outpatient clinic. To ensure privacy of the participants, entry into the room was restricted to the interviewer and the participant only. People accompanying participants were accommodated in the waiting area of the clinic. A semi-structured interview guide was prepared for these interviews exploring stress phenomenon and inquiring about life events that predisposes participants to stress with open ended questions and appropriate prompts. This was pretested in the outpatient clinics where actual sample was to be conducted. Also, the original RLCQ was shared with the participants towards the end of the interview to reflect at the listed stressors. They were encouraged to express their views over events they thought were not stressful within the context of Pakistani community which were later incorporated in the first draft. Interviews were conducted once only and participants were encouraged to share with us as much as possible with the help of prompts. Audio recording was performed. Patient identity was kept confidential. Field notes were taken during interview and were reviewed with transcription. While concluding each interview, major points shared during the interview were summarized with the participants.

Transcriptions were done of these interviews and scripts were reviewed by the first authors along with its coding. These were discussed with all the research team who reached consensus with regards to data saturation and which culminated our first draft of the adapted RLCQ (Additional file 1). It comprised of newly identified stressful events along with removal of those that were perceived as not stressful. Translation and back translation of the tool was done to ensure face validity. Later, it was sent to experts for review which included a sociologist, a psychologist and two individuals belonging to the public health domain. The experts were identified based on formal completion of post-graduate education along with greater than 10 years of local experience in the field of stress and community work in the area. Qualitative research experts also reviewed the content and layout of the work. They provided written feedback on the language, understanding, content representativeness with respect to the construct and duration for completion which culminated our second draft. This draft was pre-tested in the communities which we planned to approach for scoring items on the questionnaire (Fig. 1).

#### Step II: Scoring of items on adapted RLCQ by communities

For each item on the adapted RLCQ, we needed a score that could represent the magnitude of stress perception numerically. Hence for scoring, we required 200 participants. The sample size was based on the recommendation by Kline as a requirement of principal component analysis that we were to apply for tool development [20]. As we had 83 events on the adapted RLCQ, item to responders’ ratio of 1:2 was suggested.

Systematic random sampling technique was used in each selected area. Households were selected based on the k^th^ number that was determined from estimated population of these areas. As these communities were equally dense, we chose every eighth house based on k^th^ number. One participant was chosen at random from each household fulfilling the entry criteria. These participants were not the same from whom in-depth interviews were conducted.

Participants were asked to rate each life event in the draft on a continuum of 0 to 100 where 0 represented no stress and 100 represented maximum stress that can be tolerated.

### Human subjects approvals and registration

Ethical approval was sought from Ethical Review Committee, Aga Khan University which granted permission on 14th October 2014 with study registration ID as 3235-CHS-ERC-14. The study was registered as an observational study at Clinicaltrials.gov with the study ID NCT02356263.

### Statistical analysis plan

For statistical analysis, STATA version 12 was used. We calculated means, medians and modes for the scores of all the events in the questionnaire. We kept mean as the score of an event. In case of events in which rated mean was different from median with ±5 points, we kept median as the score of those events. We applied exploratory factor analysis on the entire data so as to classify events in the adapted questionnaire into categories reflecting similar notion.

## Results

### Step I results: Qualitative stressful life events: Themes identified

We interviewed 20 participants, equal gender with equal number of men and women with an average age of 48 years (SD = 5.6 years). 12 individuals refused to participate as they had time constraints. However, those who gave consent continued the entire interview and there were no drop outs. The mean duration of these interviews was of 57 min. Transcriptions were read multiple times by the first authors and were verified for correctness with the audio recordings. Notes were made over experiences shared by the participants. Content analysis of the data began by identifying key words and phrases from the text and segregating it into smaller units. Upon further readings, the units indicating similar experiences were coded in all the transcripts. These codes were discussed by the research team and with agreement of all, were categorized and grouped into sub-themes. Out of these sub-themes, broader themes were extracted that became the newly identified stressful life events which with final consensus of the research team were included in the adapted RLCQ. Table 2 describes the sub-themes and themes that were derived from the data along with participant’s quotes. Transcription was typed in MS word 2010. Extraction of codes and revision were first done manually then updated electronically. These themes are in congruence with actual data. The qualitative interviews conducted from participants revealed a range of stressful events that stem from the socio-political environment within Pakistan. These events described difficulties that residents face in their daily lives such as lack of power, fuel and sanitation. In addition, social beliefs and values were causes of chronic stress such as such as having an unmarried middle aged daughter, prejudices of bearing a male child, harassment in public and lack of women empowerment within homes and at work. There were references to the political environment such as state sponsored brutality, missing job opportunity because of nepotism and lack of meritocracy, extortion of money by force and suicide bombings (Table 2).

**Table 2:** Themes and sub-themes describing stressful life events of urban adult population of Karachi

Themes	Sub-themes	Participant’s Quotes
Subjected to harassment in public	• Hooting by boys in marketplaces. • Touching young females while crossing by. • Bikers speeding up towards ladies at bus stand.	• P [1]: “It is very traumatizing when one realizes that a man has touched you in public, deliberately” • P [16]: “Bikers scare us leaving unforgettable memories when they come at full speed and cross by us in public and hooting remarks on us”
Male child preference	• Giving boys freedom to go anywhere and come at whatever time without questioning at home. • Liberty to boys to go out alone. • Females are instructed to speak in lower voices which males aren’t.	• P [5]: “At home, I am usually asked where I was if I stay out of the home after sunset for giving tuitions, but never from my brother. It’s a persistent stress and threat to individuality” • P [7]: “I can’t scream even when I am in pain. I have been taught since childhood ‘girls don’t shout’. I feel inferior”
Unmarried middle aged daughter	• Due to cultural issues, worried about letting a daughter marry who is above 25 years of age. • Future insecurities about unmarried daughter while having daughter in law at home. • Monetary support for unmarried daughters while parents are retired. • Wondering about security issues of unmarried daughters of elderly parents after them.	• P [17]: “I feel anxious thinking about what my daughter’s life will be if we aren’t alive. She’s already 25. I have become obsessive in thinking how can I find my daughter a match” • P [1]: “In our society, it is difficult for lonely girls to survive. Parents retire, brothers marry and sister in laws are unpredictable. If one won’t marry, one will have to be dependent on their mood and privacy to talk to brothers”
Being a mother of an infant(s)	• Due to cultural issues, worried about letting a daughter marry who is above 25 years of age. • Future insecurities about unmarried daughter while having daughter in law at home. • Monetary support for unmarried daughters while parents are retired. • Wondering about security issues of unmarried daughters of elderly parents after them.	• P [17]: “I feel anxious thinking about what my daughter’s life will be if we aren’t alive. She’s already 25. I have become obsessive in thinking how can I find my daughter a match” • P [1]: “In our society, it is difficult for lonely girls to survive. Parents retire, brothers marry and sister in laws are unpredictable. If one won’t marry, one will have to be dependent on their mood and privacy to talk to brothers”
Being a mother of an infant(s**)**	• Difficulty in maintaining work and home responsibilities with infant. • Disturbed routine of self and added responsibilities of the infant. • Focus of family is the infant and mother feels neglected.	• P [9]: “Life with children is tough for working women. We have to meet expectations everywhere be it home, office, neighborhood” • P [11]: “I stay awake all night. I can’t have rest in the day too as I have a lot of things to handle singlehandedly. My husband thinks it is only a mother’s responsibility to take care of the kid. He is only for breadwinning”
Robbed/theft	• Mobile snatching on the streets. • Robbery at home and in banks. • Theft in public transportation such as buses and threats to kill if not surrendered.	• P [2]: “I never use my mobile phone when I am in a less busy street. I will be caught by snatchers” • P [8]: “Even in public bus, I had been a victim twice where all passengers were robbed. I had great loss from that and have stopped using buses”
Missed an opportunity because of nepotism	• Despite of capability, unable to get at a good job. • Individuals without qualification get selected because of political backing. • Easier for rich to bribe selection committee.	• P [10]: “Despite of a degree, I have lowpaid job. It is definitely suicidal for a graduate young man to be a driver, like me” • P [13]: “I have been a brilliant student, but a rich man’s son was more of a value than my talent”
Extortion or illegal demands of money by force	• To run a business safely and personal security, giving away money to political parties by force. • “Bhattas/Chanda” as locally pronounced to be given to local influential people in an area for various unrelated activities	• P [15]: “Making more money lawfully is also a trouble in this country. One has to give away it at once when demanded by influential people” • P [17]: “Nearly a quarter of my husband’s salary goes in chandas (donation) which political party people collect by force in the name of good.”
Lack of respect to females/lack of empowerment	• Not taking input from females in important decisions of the family or their own life such as marriage. • Not allowing females to attain employment or even if allowed, not outside the area of their residence. • To bear maltreatment by males in virtue of good character of a female.	• P [3]: “I am never involved in making decisions for the family despite of being eldest because I am a female” • P [7]: “When my husband bullies, I can’t even speak about it. It is not understood good in the society. A women’s character is her silence”
Being a victim of state sponsored brutality	• Due to tussles between political/influential groups, common people being abused, beaten or even killed.	• P [2]: “My father had had an accident when two local groups were fighting in public with each other. He has a disability and cannot recover”
Lack of power supplies (electricity)	• Unpredicted load shedding. • Major electrical failure by electricity providing companies. • Illegal connections built by others and lack of supply or increased cost to those who pay legally.	• P [15]: “In Karachi, electricity is a luxury. Despite paying high amount of bills, we do not get electricity for 9 h!” • P [5]: “Electrical failure follows no routine. It becomes worst in the exam days. Utility bills are high yet seldom supply. Illegal connections supply you electricity unconditionally at all times”
Lack of fuel supplies (natural gas at home, CNG, petrol)	• Limited supply of CNG in week i.e. only 3–4 days. This implies long queue when supplied. • Difficulties faced by daily wagers of public transport such as rickshaw and taxis. • Low pressure or no natural gas for cooking which implies maintaining every day routine difficult.	• P [10]: “When CNG stations are closed, passengers are lost because of lack of fuel. When stations open or partially available, it takes ages to get your turn and grab it” • P [11]: “It becomes impossible to cook food for the family in such low pressure. What could be done in half an hour takes around four hours and sometimes nothing at all!”
Insecure living environment	• Abduction for money. • Shot in robbery. • Bomb blast incidences. • Joblessness despite of qualification.	• P [15]: “I am alive today. Don’t know whether I will be kidnapped or shot dead like my friend was, just for money” • P [13]: “There is no future in Pakistan. No jobs, no security, no laws, no ethics. The world is way organized and humanitarian than it is!”
Lack of water and sanitation facilities	• Waste collection and disposal practices are poor in general. • Overflowing drains and open stagnant water creating problems for passers-by as well as to local residents.	• P [14]: “There is mixed water supply (sewerage and fresh water) in our area over an year. We complained but no use. Our kids become sick every other day” • P [12]: “In rainy season, the city becomes a mess. Drains are open, roads have loads of puddles and no one cares about it!”
Received a threat from an influential person	• Threat to life or harm to family in lieu of not doing their work or not giving them money.	• P [4]: “It has become tougher to stay safe these days. You are followed, inquired about and then threatened for well-being of family. Giving money is better than threats and torture”
Destructions due to natural disasters	• Due to floods each year, a great monetary loss is faced by the sufferers. • Improper preparation of flood plan causes many lives to be lost including cattle which are a source of living for some.	• P [12]: “There is no concept of urban planning. We make no dams despite of floods every year and later cry for a drop of clean water” • P [5]: “In our area, we expect our river to flood each year. Government makes promises which they do not keep. We lose our cattle, homes, farms and then next year thrive to build it again”
Social stigma pertinent to middle aged unmarried females	• Society considers that unmarried females do not have a good character hence no one marries them. • Stigma with respect to inability to give dowry.	• P [20]: “Society and relatives think I don’t have a good character just because I didn’t get married even by 40s” • P [3]: “My parents started gathering things to be given to me as dowry but those who come to choose me never find it enough”
Direct experience of suicide bombing	• Bomb blasts are traumatizing event both physically and psychologically. • Disabled victims bear the burden of event for lifetime. • Damage of personal property and loss of loved ones in bombing.	• P [4]: “I had witnessed a blast myself. I was praying and all of a sudden couldn’t hear anything. I was all numbed. When I recovered, all I could see was people over people. I could smell blood and flesh. To my living memory, I can’t ever forget that!”
Get to know about Suicide Bombing event on news, neighbors, city	• Stress produced by repeated coverage of the event on the television. • Fear of future of children and loved ones. • Feeling hopeless for betterment of country’s condition.	• P [9]: “I have left watching news. All it covers is disturbing. When I hear about a blast on the TV, I instantly get horrified. I rush to my phone and start calling to my family members outside just to know they are safe!” • P [2]: “It is worst to hear that a loved one has passed away in a bomb blast and when he’s so young and only one to support family”

Conversely, there was unanimous consensus among the participants to remove events such as promotion, lesser work responsibility, vacation, major increase in income, moderate purchase, major dental work, birth of a grandchild and major personal achievement. These events were regarded as “fortunate events” rather than stressful. Most participants felt that they did not have a place in the stressful life event category.

### Step II results: Categorization of events of varying stress severity by communities

The original categories of the RLCQ focus on family, personal, social, health and financial circumstances of life. It did not have a structure to take these newly identified events into their existing framework as it has no representative category that could classify environment related life stressors. We tried to do an exploratory factor analysis where each event was dealt independently so that new possible categories could be formed for the structure of adapted RLCQ. Keeping a cut-off value of ≥1of Eigen values as selection criteria of factors, we assessed 10 emerging possibilities in which all the events on the adapted draft had been grouped.

When all the events were arranged into their respective categories, they appeared to have lost the meaning of the stressors as a group because they were non-representative with each other and seemed jumbled up. Hence, we decided to place all the events in the same order of groups as it was in the original RLCQ and introduced another group of stressors having all the new events calling it as “environmental factors” (Table 3).

**Table 3:** Final draft of RLCQ

S. #	Work	LCU	My LCU	S. #	Home and family	LCU	My LCU
1.	Unwilling change to a new type of work	54		1.	Shifting within same town or city	64	
2.	Unwilling change in your work hours or conditions	54		2.	Shifting to different town, city or province	75	
3.	More work responsibilities	65		3.	Major change in living conditions	61	
4.	A demotion	69		4.	Unwilling change in family get-togethers	55	
5.	A transfer	62		5.	Major change in health or behavior of a family member	70	
6.	Trouble with your boss	57		6.	Marriage	64	
7.	Trouble with co-workers	50		7.	Pregnancy	67	
8.	Other work related difficulties	53		8.	Miscarriage or abortion	77	
9.	Major business readjustment	64		9.	Birth of a child	59	
10.	Retirement	80		10.	Adoption of a child	62	
11.	Laid off	62		11.	Relative moves in with you	64	
12.	Fired	90		12.	Spouse begins or stops work	62	
13.	Took a course to help work	52		13.	Child leaves home for marriage	79	
	Health	LCU	My LCU	14.	Child leaves home for other reasons	63	
1.	An illness or injury that kept you in bed for more than a week or sent you to hosp.	72		15.	Arguments with spouse	64	
2.	An illness or injury that was less serious than above	47		16.	Problems with relatives/in-laws	50	
3.	Major change in eating habits	46		17.	Parent’s divorce	100	
4.	Major change in sleeping habits	49		18.	A parent remarries	90	
5.	Major change in your usual type or amount of recreation	44		19.	Separation from spouse due to work	70	
	Personal and social	LCU	My LCU	20.	Separation from spouse due to marital difficulties	88	
1.	Change in personal habits	46		21.	Divorce	100	
2.	Change in school or college	53		22.	Death of a spouse	100	
3.	Change in political beliefs	48		23.	Death of a child	100	
4.	Change in religious beliefs	75		24.	Death of a parent	100	
5.	Change in social activities	51		25.	Death of a sibling	76	
6.	New, close personal relationship	51		26.	Being an elder son	77	
7.	Engagement	59		27.	Having a mentally challenged person in the family	72	
8.	Girl friend or boyfriend problems	70		28.	Dealing with a child’s chronic illnesses	77	
9.	Sexual difficulties	58			Environmental	LCU	My LCU
10.	An accident	80		1.	Robbed/theft	90	
11.	Falling out of a close personal relationship	65		2.	Missed an opportunity because of nepotism	75	
12.	Minor violation of law	52		3.	Extortion or illegal demands of money by force	90	
13.	Being held in jail	100		4.	Lack of respect to females/lack of empowerment	74	
14.	Death of a close friend	68		5.	Being a victim of state sponsored brutality	80	
15.	Subjected to harassment in public	71		6.	Lack of power supplies (electricity)	78	
16.	Male child preference	62		7.	Lack of fuel supplies (natural gas at home, CNG, petrol)	76	
17.	Unmarried middle aged daughter	76		8.	Insecure living environment	76	
18.	Being a mother of an infant(s)	60		9.	Lack of water and sanitation facilities	80	
	Financial	LCU	My LCU	10.	Received a threat from an influential person	90	
1.	Major loss of income	76		11.	Destructions due to natural disasters	90	
2.	Investment and/or credit difficulties	74		12.	Social stigma pertinent to middle aged unmarried females	85	
3.	Loss/damage to personal property	90		13.	Direct experience of suicide bombing	100	
4.	Major purchase	63		14.	Get to know about Suicide Bombing event on news, city, neighbors	76	
5.	Foreclosure or mortgage or loan	80		Total			

Out of 83, 23 events required median as event weightage whereas for others, means were closer to the median and hence we kept mean as their event weightage. At the end of this phase, we had our adapted RLCQ which was enriched with contextually relevant stressors through qualitative exploration, content validation via experts and face validity. Also, it depicts community’s perception upon severity of each event as the scores for the adapted tool were derived by community participation.

## Discussion

The adapted RLCQ enumerates stressful life events that reflect stressors within an urban population of a multiethnic city in a Low-Middle Income Country (LMIC). These events influence daily life of individuals residing in these countries such as lack of basic living facilities, gender inequality, harassment and lack of social security and were different from the original RLCQ, hence a new category of ‘environment’ was formed to incorporate these events. Moreover, the relevant communities were involved in the entire process of rating which clarified the impact of these events in people’s life.

Stressful life events for adults living in Pakistan differ from developed countries because of poverty, lawlessness and political instability. The events of our adapted RLCQ are in congruence with those that were concluded in other adaptation studies of low middle income countries [21]. It depicts that theft, lack of basic living facilities, natural disasters, social discrimination and an insecure living environment prevails in these countries and hence has been included by them in the list [22, 23]. However, being a victim of suicide bombing and state sponsored brutalities has never been highlighted in any of the studies prior to our findings. These events are important mediators of population mental health and should be part of the systematic review of health within LMIC settings.

The adapted RLCQ is a simple tool and can be administered by community health workers. It was derived from qualitative exploration following COREQ guidelines that ensure rigor within this methodology. The adapted RLCQ mirrors stressful events in context of LMICs urban population. Through community participation during rating of events exercise, we were able to understand better the importance and impact of one life event as compared to other life events for e.g. death of a family member was given a higher score as compared to death of a close friend.

There are other approaches to measuring stressful life events such as the Brown and Harris approach where contextuality of the event is discussed in detail and gives further insight [24]. However, as an initial approach, without identifying even our basic life events, Brown and Harris’s contextual approach becomes challenging as it requires great deal of expertise and time to conduct such interviews with each individual where researchers could rate the severity of stressors without being influenced with the nature and timeliness of the circumstances being explored [24]. Furthermore, considering the Pakistani context where access to community mental health facilities is difficult, identifying stress using this approach in such resource scarce setting raises concerns to its applicability [24].

The aim of the current study was to develop a community based screening tool (adapted RLCQ) rather than a sophisticated tool to measure stress in the context of Pakistani population. We have a rich resource of community health workers that is now beginning to be trained for mental health work at very basic units. From the sustainability point of view, it is much easier for community health workers to screen and identify high risk individuals based on this adapted RLCQ that consumes less time and professional expertise.

This adaptation study has certain limitations. As we interviewed participants they may have more readily shared events that they feel less stigmatized to talk about from a socio-cultural perspective. This takes into account listing stressors that are socio-culturally acceptable to discuss openly. For events that are stigmatized, such as rape, drug abuse or other such events that are social taboos, stigma would have introduced a reporting bias. Having acknowledged that, we still have noted serious issues of extortion, suicide bombing, and harassment that were reported in this study. For any country with socio-political instability, it is highly likely that new stressful events would occur in a short time frame that would affect consistency of the tool when used over longer time frames to quantify stress. Minor themes have not been discussed in this paper, as our aim of the qualitative phase was to explore stressors that reflect Pakistani population majorly so that we could include these events in our adapted tool. The applicability of the adapted RLCQ to an overseas Pakistani community becomes limited with respect to the change in environmental circumstances. Essential sociocultural values that are nurtured at early stages of life of every individual may still be applicable to Pakistanis residing outside the country however, environmental and financial stressors may differ. For instance they may face social discrimination while they may not encounter problems due to unavailability of water or electricity. Overall, the applicability of adapted RLCQ depends on the nature of the research as it becomes limited with respect to the change in environmental circumstances Future research is planned to explore the validity of the adapted RLCQ in the study population and may additionally explore resilience and resilience boosting strategies at the community level.

## Conclusions

Mental health in Pakistan needs integration in the existing infrastructure. This relevant tool can be utilized effectively in screening of individuals at the community level where effective interventions could be planned and performed for high risk groups. Initiatives such as identifying social support groups, behavioral therapies and resilience boosting strategies could help communities draw strength for coping.

## Additional file

Additional file 1: First Draft of the Adapted RLCQ. (DOCX 19 kb)

## Abbreviations

AKUH: Aga Khan University Hospital
COREQ: Consolidated Criteria for Reporting Qualitative Research
LMIC: Low-Middle Income Countries
RLCQ: Recent Life Changes Questionnaire
SRRS: Social Readjustment and Rating Scale
WHO: World Health Organization
