# Supplementary material for: Adaptation of the Recent Life Changes Questionnaire (RLCQ) to measure stressful life events in adults residing in an urban megapolis in Pakistan
Source: BMC Psychiatry. 2017 May 5;17:169. doi: 10.1186/s12888-017-1315-1 (PMC5420156; doi:10.1186/s12888-017-1315-1)
Supplement: Additional file 1: — First Draft of the Adapted RLCQ. (DOCX 19 kb) [file 12888_2017_1315_MOESM1_ESM.docx]

**Appendix 1: First Draft of the Adapted RLCQ**

| **S. No.** | **LIFE EVENTS** | **Ratings** |
| --- | --- | --- |
|  | Unwilling change to a new type of work |  |
|  | Unwilling change in your work hours or conditions |  |
|  | More work responsibilities |  |
|  | A demotion |  |
|  | A transfer |  |
|  | Trouble with your boss |  |
|  | Trouble with co-workers |  |
|  | Other work related |  |
|  | Major business readjustment |  |
|  | Retirement |  |
|  | Laid off |  |
|  | Fired |  |
|  | Took a course to help work |  |
|  | Shifting within same town or city |  |
|  | Shifting to different town, city or province |  |
|  | Major change in living conditions |  |
|  | Unwilling change in family get-togethers |  |
|  | Major change in health or behavior of a family member |  |
|  | Marriage |  |
|  | Pregnancy |  |
|  | Miscarriage or abortion |  |
|  | Birth of a child |  |
|  | Adoption of a child |  |
|  | Relative moves in with you |  |
|  | Spouse begins or stops work |  |
|  | Child leaves home for marriage |  |
|  | Child leaves home for other reasons |  |
|  | Arguments with spouse |  |
|  | Problems with relatives/ in-laws |  |
|  | Parent’s divorce |  |
|  | A parent remarries |  |
|  | Separation from spouse due to work |  |
|  | Separation from spouse due to marital difficulties |  |
|  | Divorce |  |
|  | Death of a spouse |  |
|  | Death of a child |  |
|  | Death of a parent |  |
|  | Death of a sibling |  |
|  | Being an elder son |  |
|  | Having a mentally challenged person in the family |  |
|  | Dealing with a child’s chronic illnesses |  |
|  | An illness or injury that kept you in bed for more than a week or sent you to hospital |  |
|  | An illness or injury that was less serious than above |  |
|  | Major change in eating habits |  |
|  | Major change in sleeping habits |  |
|  | Major change in your usual type or amount of recreation |  |
|  | Change in personal habits |  |
|  | Change in school or college |  |
|  | Change in political beliefs |  |
|  | Change in religious beliefs |  |
|  | Change n social activities |  |
|  | New, close personal relationship |  |
|  | Engagement |  |
|  | Girl friend or boyfriend problems |  |
|  | Sexual difficulties |  |
|  | An accident |  |
|  | Falling out of a close personal relationship |  |
|  | Minor violation of law |  |
|  | Being held in jail |  |
|  | Death of a close friend |  |
|  | Subjected to harassment in public |  |
|  | Male child preference |  |
|  | Unmarried middle age daughter |  |
|  | Being a mother of an infant(s) |  |
|  | Major loss of income |  |
|  | Investment and/or credit difficulties |  |
|  | Loss/damage to personal property |  |
|  | Major purchase |  |
|  | Foreclosure or mortgage or loan |  |
|  | Robbed/theft |  |
|  | Missed an opportunity because of nepotism |  |
|  | Extortion or illegal demands of money by force |  |
|  | Lack of respect to females/lack of empowerment |  |
|  | Being a victim of state sponsored brutality |  |
|  | Lack of power supplies (electricity) |  |
|  | Lack of fuel supplies (natural gas at home, CNG, petrol) |  |
|  | Insecure living environment |  |
|  | Lack of water and sanitation facilities |  |
|  | Received a threat from an influential person |  |
|  | Destructions due to natural disasters |  |
|  | Social stigma pertinent to middle aged unmarried females |  |
|  | Direct experience of suicide bombing |  |
|  | Get to know about Suicide Bombing event on news, neighbours, city |  |
|  | | |
